# IR Spectroscopic Degradation Study of Thin Organometal Halide Perovskite Films

**DOI:** 10.3390/molecules28031288

**Published:** 2023-01-29

**Authors:** Darkhan Yerezhep, Zhansaya Omarova, Abdurakhman Aldiyarov, Ainura Shinbayeva, Nurlan Tokmoldin

**Affiliations:** 1 Faculty of Physics and Technology, Al Farabi Kazakh National University, 71 Al-Farabi Ave., Almaty 050040, Kazakhstan; 2 Optoelectronics of Disordered Semiconductors, Institute of Physics and Astronomy, University of Potsdam, Karl-Liebknecht-Straße 24-25, 14476 Potsdam-Golm, Germany

**Keywords:** PSC, degradation, FTIR spectroscopy, functional layer, stability

## Abstract

The advantages of IR spectroscopy include relatively fast analysis and sensitivity, which facilitate its wide application in the pharmaceutical, chemical and polymer sectors. Thus, IR spectroscopy provides an excellent opportunity to monitor the degradation and concomitant evolution of the molecular structure within a perovskite layer. As is well-known, one of the main limitations preventing the industrialization of perovskite solar cells is the relatively low resistance to various degradation factors. The aim of this work was to study the degradation of the surface of a perovskite thin film CH_3_NH_3_PbI_3-x_Cl_x_ caused by atmosphere and light. To study the surface of CH_3_NH_3_PbI_3-x_Cl_x_, a scanning electron microscope, infrared (IR) spectroscopy and optical absorption were used. It is shown that the degradation of the functional layer of perovskite proceeds differently depending on the acting factor present in the surrounding atmosphere, whilst the chemical bonds are maintained within the perovskite crystal structure under nitrogen. However, when exposed to an ambient atmosphere, an expansion of the NH_3_^+^ band is observed, which is accompanied by a shift in the N–H stretching mode toward higher frequencies; this can be explained by the degradation of the perovskite surface due to hydration. This paper shows that the dissociation of H_2_O molecules under the influence of sunlight can adversely affect the efficiency and stability of the absorbing layer. This work presents an approach to the study of perovskite structural stability with the aim of developing alternative concepts to the fabrication of stable and sustainable perovskite solar cells.

## 1. Introduction

In the context of increasing energy consumption [1,2,3] and to ensure energy security and the prevention of the associated environmental damage [4,5,6], the development and rapid deployment of novel photovoltaic technologies [7,8,9], such as organometallic halide (OMH) perovskite solar cells (PSCs) [10,11,12,13,14,15], are becoming the focus of R&D activities across the world. This is a result of the unique advantages of OMH perovskites such as the ease of fabrication, high cost-effectiveness, adjustable band gap, low recombination rate, high carrier mobility and high light absorption coefficients [16,17,18,19,20,21,22,23]. During the last decade PSCs have improved their efficiency noticeably, with the cell performance achieving efficiencies in excess of 25% [24,25,26,27,28,29,30]. Improving the performance of such devices requires advanced knowledge of charge transport and dynamics within the active layer, which are directly related to the device’s efficiency. In this regard, we refer to earlier studies [31,32] which report on the investigation of a fully inorganic perovskite solar cell CsPbBr_3_. At the same time, commercialization and widespread use of PSCs are still to materialize due to the inability to maintain the device’s stability during both storage and operation [33,34,35,36].

Due to their excellent processability and good semiconductor properties, perovskite materials have shown great potential for use in microelectronics. For example, perovskite thin film field effect transistors have demonstrated low hysteresis and medium charge carrier mobility, making them highly promising for low-cost electronic applications if good stability towards external influences is achieved [37,38].

The PSC stability is affected by various degradation factors, which may be classified as internal and external. For example, the migration of perovskite ions, which affects the strength of bonds between cations and anions, is an internal degradation factor [39,40,41,42,43]. Therewith, the preservation of the crystal structure and the stoichiometric ratio of the PSC components may be attained via the suppression of ion migration [43,44,45]. External factors include environmental influences such as humidity [46,47,48], oxygen [49,50,51], temperature [52,53,54] and radiation [49,55,56,57]. The intensive impact of the environment leads to a decrease in the strength of the CH_3_NH_3_^+^ hydrogen bond, which destroys the PSC structure [58,59]. For example, degradation of PSCs upon adsorption of water molecules may occur via a strong distortion of the interatomic distance [60]. In order to avoid exposure to humidity and oxygen, a significant effort is placed on the development of effective encapsulation solutions [61,62,63,64]. Thus, the problem of stability leads to suspended commercialization of perovskite photovoltaics, which is already competitive with other photovoltaic technologies in terms of PCE. One approach to address this was shown in Reference [65], which describes the fabrication of perovskite layers with different band gaps by changing the ratio of bromine (Br) to iodine (I) in the position of the halide anion. Here, the authors report the creation of a wide-bandgap PSC using an inexpensive inorganic transport layer, which provides improved efficiency and remarkable stability.

Some of the successful examples enable the achievement of excellent PSC temperature cycling stability (−40 to 85 °C), with more than 90% optoelectronic performance remaining after 200 temperature cycles and more than 1000 h of operation [66,67,68,69]. For example, Reference [70] shows that perovskite lattice deformation resulting from extreme temperature fluctuations (from −160 to 150 °C) can be restored.

In spite of such success, the influence of temperature on the stability of perovskites under atmospheric conditions and when exposed to light is not fully understood. At an early stage in the development of perovskite solar cells, there was already an awareness of the dramatic role of humidity in the deterioration and degradation of the initial characteristics of a solar cell [71]. Perovskite thin films, compared with single crystals, are subject to faster degradation due to the greater number of grain boundaries, which ensures rapid water penetration. Water molecules are quite easily included in the perovskite lattice due to their ability to form hydrogen bonds with lattice iodides, which leads to destruction. Narrowly focused experimental studies are required to establish the hydrate phase and its effect on the optical properties of a thin perovskite film.

Understanding the underlying degradation mechanisms is essential to improve the performance, sensitivity and stability of organometallic perovskite devices. To study the mechanisms of degradation and thermal instability in PSCs, spectroscopic analyses are usually required. These include techniques such as X-ray diffraction [72,73], photoelectron spectroscopy [74,75], thermogravimetric analysis [76,77], mass spectrometry [78,79], scanning electron microscopy [80,81] and Fourier transform infrared (FTIR) spectroscopy [82,83,84,85,86]. 

In this paper, we present the results of a study of the evolution of the crystal structure of a separate perovskite layer under the influence of the surrounding atmosphere. The degradation of the surface structure of the perovskite layer was studied using scanning electron microscopy. Using IR spectroscopy, the vibrational spectra of the samples were measured under various conditions. The absorption spectra of the samples in the wavelength range of 300–1100 nm were obtained using a QEX10 automated setup. The obtained results indicate that the perovskite crystal structure degrades differently depending on external influences. It can be assumed that under the influence of the atmosphere, the surface layers are mainly subjected to degradation, whereas under the influence of light, volumetric degradation of the perovskite occurs.

## 2. Results and Discussion

The susceptibility of perovskites to the external environment leads to the combined impact of factors such as moisture, oxygen and lighting, which leads to a deterioration of the photovoltaic characteristics and an accelerated degradation. Exposure to an environment with relative atmospheric humidity above 50% leads to the formation of hydrogen bonds with organic perovskite cations, where the perovskite easily interacts with water molecules, ultimately creating a hydrated product (CH_3_NH_3_)_4_PbI_6_·2H_2_O [87,88,89]. In this case, degradation of the perovskite structure and a decrease in the absorption of the spectrum in the visible region are observed, and faster destruction of the perovskite occurs due to a weak chemical bond between the octahedral framework and the weakly bonding cation. It was noted in [90] that perovskite decomposes quite quickly with the release of a yellow substance PbI_2_ due to the interaction with water, whereby water protonates iodide, leading to the appearance of hydrogen iodide (HI). In order to reduce the harmful effect of oxygen and moisture on the perovskite stability, the control the iodide defects is crucial [91]. This paper considers the evolution of the chemical structure of an individual functional perovskite layer using the FTIR spectroscopy, SEM and optical absorption measurements. In the work, samples were studied in an amount from 10 to 50 in order to ensure reproducibility of the results.

### 2.1. FTIR

This section presents the results of IR spectroscopic studies of the degradation of the perovskite films under the influence of an ambient environment and visible illumination in the wavelength range of 380–800 nm. IR spectroscopy was used previously to study the evolution of the chemical structure of individual functional layers and their combinations under the influence of the surrounding atmosphere and temperature [83,92,93]. The spectral measurement range in our study was 600–4200 1/cm.

The process of atmospheric degradation was studied by placing the resulting perovskite film inside a glove box in the dark at room temperature T = 295 K. The film was kept in the glove box for 600 h. For statistics, 10 samples were selected to assess degradation from the total of 50 manufactured films. The procedure for testing the stability of the samples partially complies with the ISOS-D-1 protocol [94].

**Figure 1:**
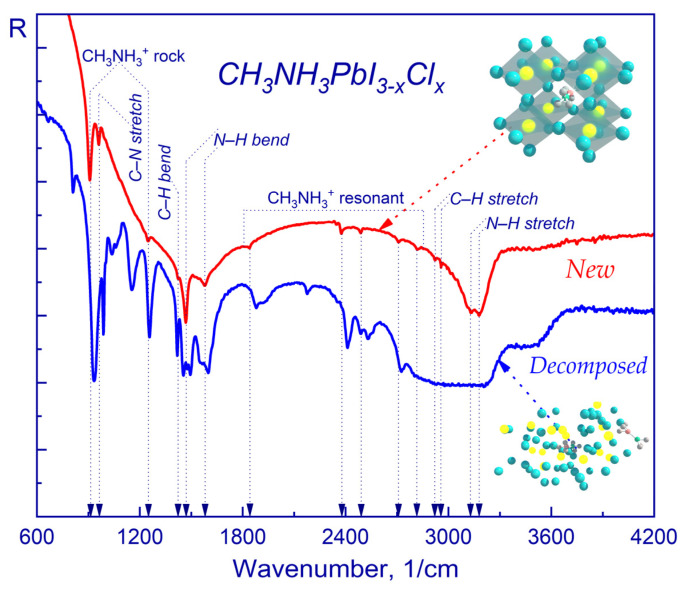
Fourier transform IR spectra of CH_3_NH_3_PbI_3-x_Cl_x_ single crystal samples: freshly prepared (red) perovskite thin film and exposed to atmosphere (blue).

Figure 1 shows the vibrational spectra employed for identifying the evolution of the chemical structure using characteristic transmission bands recorded at a high signal-to-noise ratio and in the wavenumber range of 400–4200 1/cm from a ~700 nm thick CH_3_NH_3_PbI_3-x_Cl_x_ sample before and after the sample degradation. Based on the published data [80,83,85,86,92,93,95,96,97,98,99,100], a good convergence of the vibrational spectra was observed when compared with the experimentally obtained vibrational spectra (red line) of a freshly obtained (new) sample. In a freshly produced thin perovskite film, the most intense vibrational mode was observed at the frequencies of 3132 1/cm and 3179 1/cm, which corresponded to the symmetric and asymmetric N-H stretching modes (associated with NH_3_^+^). It should be noted that there was no visible feature, corresponding to O–H stretching vibrations in the region of 3400–3700 1/cm, which indicated the presence of a functional hydroxyl group (hydrates, hydroxide and water) in the freshly obtained thin perovskite film [97,101,102].

The following peaks are typical for a freshly prepared perovskite film: 910 1/cm and 1248 1/cm (CH_3_NH_3_^+^ rock), 961 1/cm (C–N stretch), 1421 1/cm (C–H bend), 1468 1/cm (N–H bend (symmetric)), 1578 1/cm (N–H bend (asymmetric)), 2921 1/cm (C–H stretch (symmetric)) and 2958 1/cm (C–H stretch (asymmetric)).

Exposure of the perovskite film to the ambient environment was conducted at relative humidity of 50% ± 5%. It is known, that when exposed to the atmosphere, the NH_3_^+^ band widens, which is accompanied by a shift of the N–H stretching mode toward higher frequencies (blue line). The simultaneous expansion and shift of the N–H vibrational peak at ~3200 1/cm may be due to the degradation of the perovskite film via its hydration. Another sensitive characteristic of the hydration process was the appearance of new infrared absorption peaks at 1660 1/cm and 1497 1/cm [97]. The dominant peak at 1660 1/cm represented the bending mode of the N–H and O–H bonds, while the peak at 1497 1/cm was due to the stretching of the O–H and C–H bonds [93]. Additionally, we observed spectral shifts due to the destruction of the crystalline structure in the region of 900 1/cm–1300 1/cm: the peaks at 910 1/cm and 1248 1/cm (CH_3_NH_3_^+^ rock) shifted to 935 1/cm and 1255 1/cm, respectively, whereas the peak at 961 1/cm (C–N stretch) shifted to 990 1/cm. Hence, it is evident that the hydration leads to the formation of new chemical bonds within the perovskite crystal structure due to the deprotonation of CH_3_NH_3_^+^ [80].

Next, the stability of the samples to light exposure was studied using an LED lamp. The illuminance of the samples was controlled using a Digisense 20250-00 light meter, calibrated according to the NIST standard, before each measurement. The characteristics of the LED lamp were as follows: brand—OSRAM Parathom Classic P25, electric power—4 W, light flux—250 lm and wavelength range—380 to 800 nm. The exposure was carried out entirely in an inert atmosphere (N_2_) and without access to external lighting. Nitrogen with a purity of 99.999% was used to create an inert environment in accordance with ISO 2435-73, while the atmospheric condition in the glove box corresponded to ISO 106482:1994 (oxygen concentration ~0.4 ppm and humidity ~2 ppm). Thus, the samples were illuminated for 30 days. Then, the samples were taken without encapsulation to the ambient environment, and optical studies were carried out immediately to evaluate degradation by exposure to light in the indicated wavelength range.

Photodegradation proceeded via the decomposition of CH_3_NH_3_PbI_3_ into PbI_2_ and CH_3_NH_3_I, followed by further decomposition of the latter product into CH_3_N_2_ and I_2_ [103]. Therefore, three stages of the photodegradation process have been proposed [57]. During the first stage of photodegradation of CH_3_NH_3_PbI_3-x_Cl_x_, volatile gases such as CH_3_I, NH_3_ and PbI_2_ were formed. In the next step, a reversible reaction took place, which led to the formation of CH_3_NH_3_, HI and PbI_2_. The final step resulted in PbI_2_ forming Pb_0_ and volatile I_2_ [104]. Accordingly, iodine vacancies and HI were formed on the surface of the perovskite crystal or at the grain boundary due to the deprotonation of CH_3_NH_3_^+^. This led to further decomposition of perovskite. CH_3_NH_3_PbI_3_, due to the chain chemical reaction of decomposition of iodine and PbI_2_ in the perovskite structure, can irreversibly photodegrade with the formation of lead and iodine, which contributed to the destruction of the perovskite crystal structure. In Reference [105], the authors aimed at reversing the photodegradation of CH_3_NH_3_PbI_3_ by adding gaseous CH_3_NH_2_ in a glove box with the sample; however, this approach could not establish a continuous recovery cycle due to the gradual loss of I. Overall, a detailed understanding of the variations in the chemical components of perovskite during exposure to light is still in its initial state and further research is needed to improve the device’s stability.

**Figure 2:**
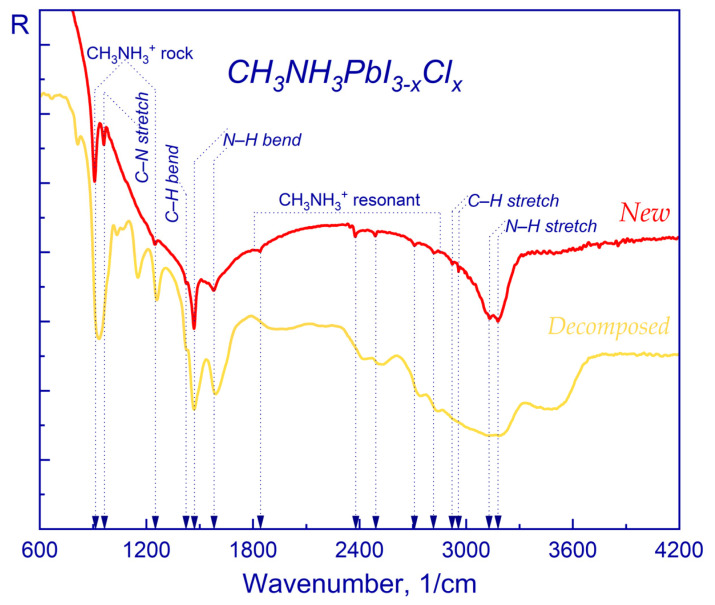
IR spectra of CH_3_NH_3_PbI_3-x_Cl_x_: freshly prepared (red) and exposed to illumination (yellow).

FTIR spectroscopy was used to investigate the influence of light exposure, as it is sensitive to the presence of hydroxyl groups, which play an important role during the photovoltaic process by restraining injection and charge transfer, as well as energy transfer from the perovskite crystallites to OH vibrational states [106,107]. Figure 2 shows a comparison of the IR spectra of a freshly prepared sample (new) with a degraded (decomposed) sample under the influence of the LED lamp. 

Comparison of the IR spectra of the freshly prepared and degraded samples demonstrates, that the intensity of the band, corresponding to the stretching vibrations of hydroxyl groups, decreases. This may indicate desorption or dissociation of the hydroxyl groups within the sample. Accordingly, the dissociation of H_2_O molecules under the influence of light can adversely affect the efficiency and stability of the absorbing layer due to the formation of defects. A more pronounced peak, corresponding to N–H bending (asymmetric) vibrations is observed with a shift in position from 1578 1/cm to 1590 1/cm. One can also see pronounced peaks at 936 1/cm and 1260 1/cm that belong to CH_3_NH_3_^+^ rocking oscillations, which indicates degradation of the perovskite film. Preservation of the efficiency and stability of a perovskite solar cell can be achieved by passivating ionic defects, i.e., by increasing the recombination lifetime and reducing the density of charge traps [108,109].

The presented results indicate a rapid degradation of the perovskite film when exposed to an ambient environment (atmosphere and light). It was also observed that in some cases the degraded film had a grey–yellow tint after degradation, which is unusual and not similar to the bright yellow color of the PbI_2_ films—a typical material residue after degradation.

**Figure 3:**
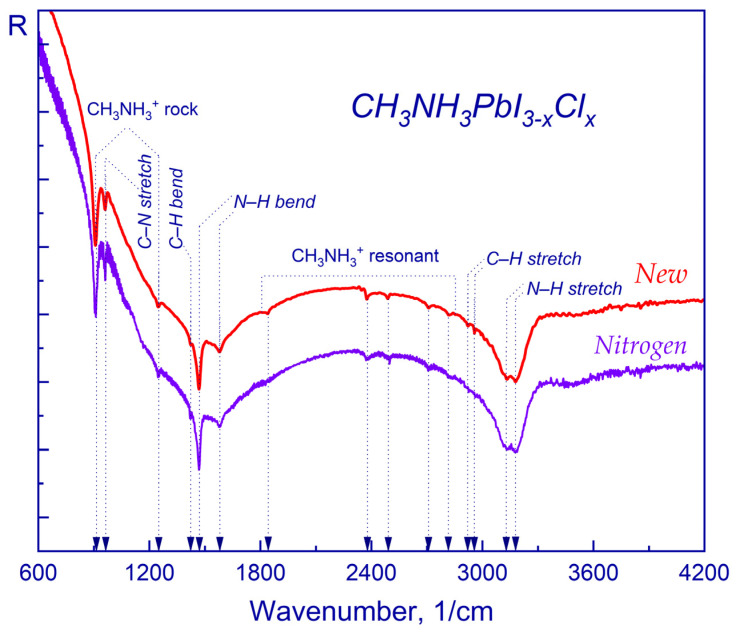
Fourier transform IR spectra of CH_3_NH_3_PbI_3-x_Cl_x_: freshly prepared (red) and exposed to nitrogen (purple).

It can be seen that different types of environmental influences, be it atmosphere or light, lead to different routes of decomposition of the perovskite crystal structure. In order to maintain efficiency and avoid degradation, the impact of a nitrogen environment on the surface of a thin perovskite film was studied. The results of the study are shown in Figure 3, where the main characteristic vibrations of the CH_3_NH_3_PbI_3-x_Cl_x_ band are identified.

It is clear that the exposure of the perovskite film in a nitrogen environment does not lead to any significant changes, including the CH_3_ vibration band at 910 1/cm and the scissoring of the CH vibrational band at 1468 1/cm of the CH_3_ functional group. When comparing a freshly obtained thin perovskite film with an aged sample in a nitrogen atmosphere, oscillations were observed at frequencies equal to 3132 1/cm and 3179 1/cm. The absence of the functional hydroxyl group feature in the region of 3400–3700 1/cm, which corresponds to the O–H stretching vibrations, confirms the preservation of the crystal structure of the sample under study. Thus, we conclude that the original properties of a thin perovskite film are preserved in the nitrogen environment.

Our results largely correlate with those reported by other authors [110]. However, in the previous study, no degradation of the perovskite sample under the influence of light in the visible range and in the absence of oxygen was reported. In contrast, in our case, the sample underwent degradation under the influence of light. This could be related to the accepted standard conditions (ISO 106482:1994), as well as the difference in the sample compositions (CH_3_NH_3_PbI_3-x_Cl_x_ vs. CH_3_NH_3_PbI_3_ [110]), which may affect film morphology [111] and, respectively, stability.

### 2.2. Surface Morphology

**Figure 4:**
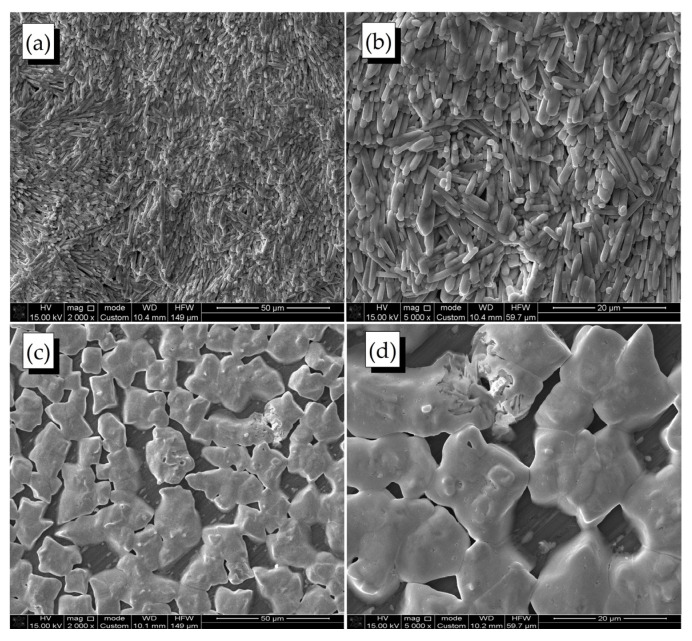
Scanning electron microscope images of the perovskite films spin-coated at different speeds (before and after atmospheric degradation): (**a**) before, (magnification 2000×); (**b**) before, (magnification 5000×); (**c**) after atmospheric degradation, (magnification 2000×); (**d**) after atmospheric degradation, (magnification 5000×).

Figure 4 shows micrographs of methylammonium iodide-lead chloride perovskite films deposited on a crystalline silicon substrate at spin speed 1000 rpm. 

Figure 4 demonstrates that the surface of the perovskite films has a branched structure, with the size of the crystallites being sensitive to the speed of spin-coating. The absence of pores or voids, nevertheless, indicates a good quality of the films, which is critical for the photovoltaic application. Initially, no significant changes were observed on the surface of the degraded film; however, with prolonged exposure to the atmosphere (more than 600 h), decomposition and destruction of the surface of the perovskite film could be noticed (see Figure 4c,d).

### 2.3. Optical Density

**Figure 5:**
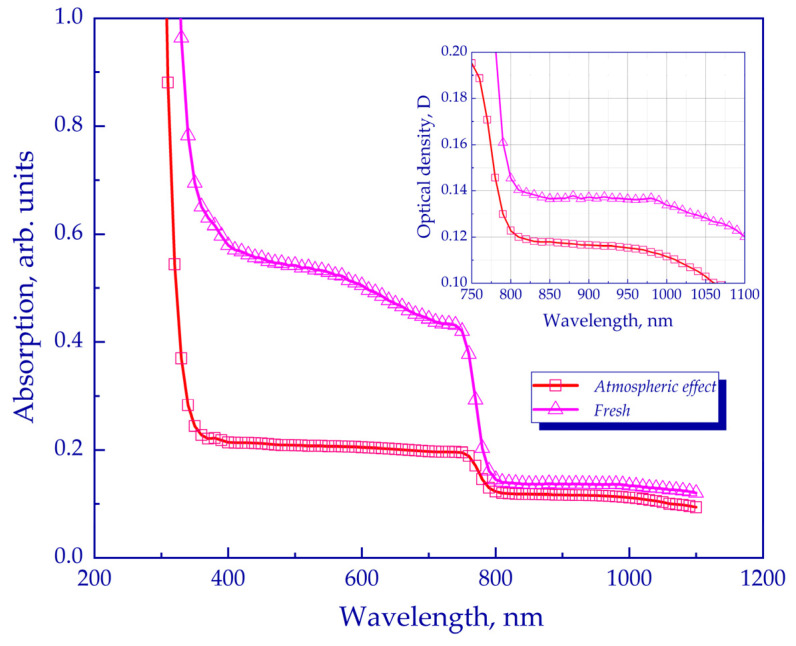
Variation in the absorption of perovskite films due to various factors.

The evaluation of the optical characteristics of the degraded perovskite films was carried out using optical absorption spectroscopy. Weakening of the absorption of the absorption spectra was observed under prolonged exposure to the ambient atmosphere, which caused the degradation of the sample and which led to the release of a yellow substance PbI_2,_ as well as a decrease in the absorption intensity. The optical absorption through the sample films was measured in the range from 300 nm to 1100 nm. The obtained spectra of degraded films under the influence of the atmospheric factor are shown in Figure 5. 

Upon degradation under the influence of the atmospheric factor, a stronger decrease in the light absorption can be observed in the visible region of 400–750 nm. Thus, the obtained experimental data demonstrate the impact of degradation on the optical properties of the perovskite film. 

## 3. Materials and Methods

### 3.1. Materials

N,N-dimethylformamide (HCON(CH_3_)_2_, 99.8%, Sigma-Aldrich, UK), methylammonium iodide (CH_6_IN, >99.5%, LumTec, Taiwan) and lead(II) chloride (PbCl_2_, 99.999%, LumTec, Taiwan) were used as is to obtain the perovskite layers. The chemicals were used without any further purification. The resulting solution was deposited on glass substrates coated with tin oxide (FTO) (Solaronix SA, Switzerland), as well as on n-doped silicon substrates (200 μm, Atecom, Taiwan). The substrates were preliminarily cleaned using distilled water, acetone, ethanol and Hellmanex. After washing, the substrates were dried in a muffle furnace at a temperature of 100 °C for an hour. The choice of a crystalline silicon substrate was related to its high transparency in the IR range. This made it possible to conduct an IR spectroscopic study of the perovskite structure of the film in a nondestructive manner.

### 3.2. Preparation of CH3NH_3_PbI_3-x_Cl_x_

**Figure 6:**
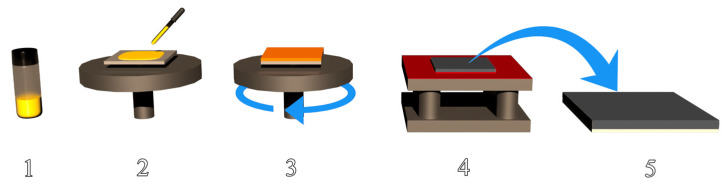
Schematic illustration of the perovskite thin film preparation: 1—dissolution of CH_3_NH_3_PbI_3-x_Cl_x_, 2—application of 50 µL solution to the substrate, 3—spin coating at 1000 rpm for 30 s, 4—annealing at 100–120 °C for 100 min, 5—resulting perovskite sample.

The CH_3_NH_3_PbI_3-x_Cl_x_ perovskite films were fabricated in an inert atmosphere at room temperature T = 295 K. To ensure better dissolution of the solid precursors CH_3_NH_3_I and PbCl_2_ in N, N-dimethylformamide at the total concentration of 0.66 g/mL, the vial was heated at 60 °C. The weight ratio of 3:1 between CH_3_NH_3_I and PbCl_2_ was used. The samples were prepared via spin-coating at 1000 rpm for 30 s, then left to dry for 10 min, followed by annealing using a C-MAG HP 7 hotplate (IKA, Staufen, Germany) at 100 °C for 90 min, and further at 120 °C for 10 min. The prepared samples were left to cool down to room temperature in an inert atmosphere. Individual perovskite layers had an approximate thickness of 700 nm. A scheme of the process is shown in Figure 6, and follows a recipe described elsewhere [112]. 

### 3.3. Film Characterization

The morphology of the samples was studied using scanning electron microscopy (Quanta 200i 3D, FEI Company, Hillsboro, OR, USA). The optical properties were measured using a QEX-10 quantum efficiency measurement tool (PV Measurements, Inc., Boulder, CO, USA). The vibrational spectra were recorded using an INFRASPEK FSM 2203 FTIR spectrometer in the spectral range of 370–7800 1/cm with a maximum resolution of 0.125 1/cm and a signal-to-noise ratio exceeding 60,000.

## 4. Conclusions

Based on the vibrational spectroscopy studies on the stability of the CH_3_NH_3_PbI_3-x_Cl_x_ perovskite films, the following conclusions can be drawn. Strong changes in the absorption intensity of the characteristic frequencies corresponding to the NH and CH functional groups were revealed. In the range of the stretching vibrations of these groups, intense vibrational modes at the frequencies of 3132 1/cm and 3179 1/cm for the as-prepared and aged films differed in terms of the width of the bands. We attribute these differences to the degradation of the perovskite structure. This is confirmed by SEM and absorption spectroscopy studies. The surface morphology of the samples varied greatly under the influence of the atmosphere and light, which led to the destruction of the perovskite layer on the substrate as a whole. Accordingly, this destruction was accompanied by hydration degradation of the crystal structure with the formation of new chemical bonds via the deprotonation mechanism. The results of this study, as well as previous results by other authors [83,92,93], suggest that the exposure of the perovskite to the natural environment leads to the breaking of iodide bonds in its crystalline structure. In principle, the degradation dynamics of perovskite structures can be heterogeneous and varied, and we hope that this work will provide a lens through which studies on the stability of perovskite solar cells can be viewed. Understanding the stability limitations of organohalide perovskite films is hoped to eventually bring the perovskite photovoltaic technology closer to the competitive thin film photovoltaic industry.

## Data Availability

Data will be made available upon a reasonable request to the corresponding author.

## Sample Availability

Samples of the compounds are available from the organization upon reasonable request.
